# Dynamics of online hate and misinformation

**DOI:** 10.1038/s41598-021-01487-w

**Published:** 2021-11-11

**Authors:** Matteo Cinelli, Andraž Pelicon, Igor Mozetič, Walter Quattrociocchi, Petra Kralj Novak, Fabiana Zollo

**Affiliations:** 1 Ca’ Foscari University of Venice, Venice, Italy; 2 Jozef Stefan Institute, Ljubljana, Slovenia; 3 Jozef Stefan International Postgraduate School, Ljubljana, Slovenia; 4 Sapienza University of Rome, Rome, Italy

**Keywords:** Computer science, Information technology

## Abstract

Online debates are often characterised by extreme polarisation and heated discussions among users. The presence of hate speech online is becoming increasingly problematic, making necessary the development of appropriate countermeasures. In this work, we perform hate speech detection on a corpus of more than one million comments on YouTube videos through a machine learning model, trained and fine-tuned on a large set of hand-annotated data. Our analysis shows that there is no evidence of the presence of “pure haters”, meant as active users posting exclusively hateful comments. Moreover, coherently with the echo chamber hypothesis, we find that users skewed towards one of the two categories of video channels (questionable, reliable) are more prone to use inappropriate, violent, or hateful language within their opponents’ community. Interestingly, users loyal to reliable sources use on average a more toxic language than their counterpart. Finally, we find that the overall toxicity of the discussion increases with its length, measured both in terms of the number of comments and time. Our results show that, coherently with Godwin’s law, online debates tend to degenerate towards increasingly toxic exchanges of views.

## Introduction

Public debates on social media platforms are often heated and polarised^1–3^. Back in the 90s, Mike Godwin coined a theorem, today known as Godwin’s law, stating that “As an online discussion grows longer, the probability of a comparison involving Nazis or Hitler approaches to one”. More recently, with the advent of social media, an increasing number of people is reporting exposure to online hate speech^4^, leading institutions and online platforms to investigate possible solutions and countermeasures^5^. To prevent and counter the spread of hate speech online, for example, the European Commission agreed with Facebook, Microsoft, Twitter, YouTube, Instagram, Snapchat, Dailymotion, Jeuxvideo.com, and TikTok on a “Code of conduct on countering illegal hate speech online”^6^. In addition to fuelling the toxicity of the online debate, hate speech may have severe offline consequences. Some researchers hypothesised a causal link between online hate and offline violence^7–9^. Furthermore, there is empirical evidence that online hate may induce fear of offline repercussions^10^. However, the detection and contrast of hate speech is complicated. There are still ambiguities in the very definition of hate speech, with academic and relevant stakeholders providing their own interpretations^4^, including social media companies such as Facebook^11^, Twitter^12^, and YouTube^13^.

We use the term “hate speech” to cover whole spectrum of language used in online debates, from normal, acceptable to the extreme, inciting violence. On the extreme end, violent speech covers all forms of expression which spread, incite, promote or justify racial hatred, xenophobia, antisemitism or other forms of hatred based on intolerance, including: intolerance expressed by aggressive nationalism and ethnocentrism, discrimination and hostility against minorities, migrants and people of immigrant origin^14^. Less extreme forms of unacceptable speech include inappropriate (e.g., profanity) and offensive language (e.g., dehumanisation, offensive remarks), which is not illegal, but deteriorates public discourse and can lead to a more radicalised society.

In this work, we analyse a corpus of more than one million comments on Italian YouTube videos related to COVID-19 to unveil the dynamics and trends of online hate. First, we manually annotate a large corpus of YouTube comments for hate speech, and train and fine-tune a hate speech deep learning model to accurately detect it. Then, we apply the model to the entire corpus, aiming to characterise the behaviour of users producing hate, and shed light on the (possible) relationship between the consumption of misinformation and usage of hate and toxic language. The reason for performing hate speech detection on the Italian language is two-fold: First, Italy was one of the countries most affected by the COVID-19 pandemic and especially by the early application of non-pharmaceutical interventions (strict lockdown happened on March 9, 2020). Such an event, by forcing people at home, increased the internet use and was likely to exacerbate the public debate and foment hate speech against specific targets such as the government and politicians. Second, Italian is a less studied language in comparison to English or German^15^ and, to the best of our knowledge, this is the first study to investigate hate speech in Italian on YouTube.

This work advances the current literature at different levels. There is a large body of literature about community-level hate speech^16–18^. However, less is known about the behavioural features of users using hate speech on mainstream social media platforms, with few recent exceptions for Twitter^19–21^ and Gab^18^. Furthermore, to our knowledge, the relationship between online hate and misinformation is yet to be explored. In this paper, we study hate speech with respect to a controversial and heated topic, i.e., COVID-19, which has been already analysed in terms of sinophobic attitudes^22^. We relax the assumption behind many community-based studies, for which every post produced within an online community hosting haters is hate^17,23^. Instead, to cope with a classification task that involves more than one million comments, we annotate a high-quality dataset of more than 70,000 YouTube comments, which is used for training and evaluating a deep learning model. The model is standard in the state-of-the-art and builds on a wide strand of literature using machine learning^24–26^ and deep learning^27–29^ for automatic hate speech detection via text classification. Moreover, we distinguish YouTube channels into two categories: questionable, i.e., channels likely to disseminate misinformation, and reliable. This categorisation is in line with previous studies on the spreading of misinformation^30–32^, and builds on a list of misinformation sources provided by the Italian Communications Regulatory Authority (AGCOM).

Our results show that hate speech on YouTube is slightly more present than on other social media platforms^20,21,33^ and that there are no significant differences between the proportions of hate speech detected in comments on videos from questionable and reliable channels. We also note that hate speech does not show specific temporal patterns, even on questionable channels. Interestingly, we do not find evidence of “pure haters”, intended as active users posting exclusively hateful comments. Still, we note that users skewed towards one of the two categories of video channels (questionable, reliable) are more prone to use toxic language—i.e., inappropriate, violent, or hateful—within their opponents community. Interestingly, users skewed towards reliable content use on average a more toxic language than their counterpart. Finally, we find that the overall toxicity of the discussion increases with its length measured both in terms of the number of comments and time. In other words, online debates tend to degenerate towards increasingly toxic exchanges of views, in line with Godwin’s law.

## Methods

### Data collection

We collected about 1.3M comments posted by more than 345,000 users on 30,000 videos from 7000 channels on YouTube. According to summary statistics about YouTube by Statista^34^, the number of YouTube users in 2019 in Italy was about 24 millions (roughly one third of the Italian population). By applying 1% empirical law, for which in an Internet community 99% of the participants just visualise content (the so-called lurkers), while only 1% of the users actively participate in the debate (e.g., interacting with content, posting information, commenting), we can evaluate the representativeness of our dataset. Therefore, we can expect that, out of 24 millions users on the platform, a population of 240,000 users usually interact with the content. Taking into account these estimates, the size of our sample (345,000) seems to be appropriate, especially when considering that we are focusing on a specific topic (COVID-19) and not on the whole content of the platform. These considerations are also consistent with another statistic of our dataset, where the videos show an average of 5M daily views (with peaks at 20M).

Using the official YouTube Data API, we performed a keyword search for videos that matched a list of keywords, i.e., {*coronavirus, nCov, corona virus, corona-virus, covid, SARS-CoV*}. An in-depth search was then performed by crawling the network of related videos as provided by the YouTube algorithm. Then, we filtered the videos that matched our set of keywords in the title or description from the gathered collection. Finally, we collected the comments received by these videos. The title and the description of each video, as well as the comments, are in Italian according to the Google’s cld3 language detection service. The set of videos covers the time window that goes from 01/12/2019 to 21/04/2020, while the set of comments ranges in the time window that goes from 15/01/2020 to 15/06/2020.

We assigned a binary label to each YouTube channel to distinguish between two categories: questionable and reliable. A questionable YouTube channel is a channel producing unverified and false content or directly associated to a news outlet that failed multiple fact checks performed by independent fact checking agencies. The list of YouTube channels labelled as questionable was provided by the Italian Communications Regulatory Authority (AGCOM). The remainder of the channels were labelled as reliable. Table 1 shows a breakdown of the dataset.

**Table 1:** Breakdown of YouTube data.

	Channels	Videos	Comments
**Category**			
Reliable	7140	29,975	1,170,461
	99.7 %	98.5 %	91.8 %
Questionable	17	464	103,475
	0.3 %	1.5 %	8.2 %
Total	7157	30,436	1,273,930
	100 %	100 %	100 %

### Hate speech model

Our aim is to create a state-of-the-art hate speech model, by deep learning methods. We first produce two high-quality manually annotated datasets for training and evaluating the model. The training set is intentionally selected to contain as much hate speech vocabulary as possible, while the evaluation set is unbiased, to assure proper model evaluation. We then apply the model to all the collected data and study the relationship between the hate speech phenomenon and misinformation.

Deep learning models based on Transformer architecture outperform other approaches to automated hate speech detection, as evident from recent shared tasks in the SemEval-2019 evaluation campaign: HatEval^28^ and OffensEval^35^, as well as OffensEval 2020^29^. The central reference for hate speech detection for Italian is the report on the EVALITA 2018 hate speech detection task^36^. Furthermore, in^37^ authors modelled the hate speech task using the Italian pre-trained language model AlBERTo, achieving state-of-the-art results on Facebook and Twitter datasets. We trained a new hate speech detection model for Italian following the state-of-the-art approach^37^ on our four-class hate speech detection task (see sections “Data selection and annotation” and “Classification” for detailed information).

#### Data selection and annotation

The comments to be annotated were sampled from the Italian YouTube comments on videos about the COVID-19 pandemic in the period from January 2020 to May 2020. Two sets were annotated: a hate-speech-rich training set with 59,870 comments and an unbiased evaluation set with 10,536 comments.

To get a *training set* that is rich with hate speech, we annotated all the comments with a (basic) hate speech classifier (machine learning model) that assigns a score between -3 (hateful) and +3 (normal). The basic classifier was trained on a publicly available dataset of Italian hate speech against immigrants^38^. Even though this basic model is not very accurate, its performance is better than random and we used its result for selecting the training data to be annotated and later used for training our deep learning model. For a realistic evaluation scenario, threads (i.e., all the comments to the video) were kept intact during the annotation procedure, yet individual comments were annotated.

The threads (with comments) to be annotated for the *training set* were selected according to the following criteria: thread length (the number of comments in a thread between 10 and 500), and hatefulness (at least 5% of hateful comments according to our basic classifier). The application of these criteria resulted in 1168 threads (VideoIds) and 59,870 comments. The *evaluation set* was selected from May 2020 data as a random (unbiased) sample of 151 threads (VideosIds) with 10,543 comments.

Our hate speech annotation schema is adapted from OLID^39^ and FRENK^40^. We differentiate between the following speech types:

- Acceptable (non hate speech);
- Inappropriate (the comment contains terms that are obscene or vulgar, but the text is not directed to any person or group specifically);
- Offensive (the comment includes offensive generalisation, contempt, dehumanisation, or indirect offensive remarks);
- Violent (the comment’s author threatens, indulges, desires or calls for (physical) violence against a target; it also includes calling for, denying or glorifying war crimes and crimes against humanity).

The data was split among eight contracted annotators. Each comment was annotated twice by two different annotators. The splitting procedure was optimised to get approximately equal overlap (in the number of comments) between each pair of annotators for each dataset. The annotators were given clear annotation guidelines, a training session and a test on a small set to evaluate their understanding of the task and their commitment before starting the annotation procedure. Furthermore, the annotation progress was closely monitored in terms of the annotator agreement to ensure high data quality.

**Figure 1:**
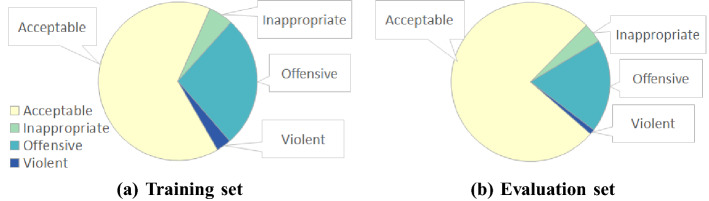
The distribution of the four hate speech labels in the manually annotated training (**a**) and evaluation (**b**) sets. The training set is intentionally biased to contain more hate speech while the evaluation set is unbiased.

The annotation results for the training and evaluation sets are summarised in Fig. 1. The annotator agreement in terms of Krippendorff’s $Alpha$ ^41^ and accuracy (i.e., percentage of agreement) on both the training and the evaluation sets is presented in Table 2. The agreement results indicate that the annotation task is difficult and ambiguous, as the annotators agree on the label in only about 80% of the cases. Since the class distribution is very unbalanced, accuracy is not the most appropriate measure of agreement. $Alpha$  is a better measure of agreement as it accounts for the agreement by chance. Our agreement scores in terms of $Alpha$  are comparable to those of other high-quality datasets, like^21,42^.

**Table 2:** The annotation results for the training and evaluation datasets: date range, size (no. of comments annotated twice), and the annotator agreement in terms of interval Krippendorff’s $Alpha$  and accuracy ($Acc$).

Dataset	Dates	Size	$Alpha$	$Acc$
Training	Jan.–Apr. 2020	59,870	0.59	0.77
Evaluation	May 2020	10,543	0.56	0.82

#### Classification

A state-of-the-art neural model based on Transformer language models was trained to distinguish between the four hate speech classes. We use a language model based on the BERT architecture^43^ which consists of 12 stacked Transformer blocks with 12 attention heads each. We attach a linear layer with a softmax activation function at the output of these layers to serve as the classification layer. As input to the classifier, we take the representation of the special [CLS] token from the last layer of the language model. The whole model is jointly trained on the downstream task of four-class hate speech detection. We used AlBERTo^44^, a BERT-based language model pre-trained on a collection of tweets in the Italian language. According to previous work^43^, fine-tuning of the neural models was performed end-to-end. We used the Adam optimizer with the learning rate of $2e-5$ and learning rate warmup over the first 10% of the training instances. We used weight decay set to 0.01 for regularization. The model was trained for 3 epochs with batch size 32. We performed the training of the models using the HuggingFace Transformers library^45^.

The tuning of our models was performed by cross validation on the training set, while the final evaluation was performed on the separate out-of-sample evaluation set. In our setup, each data instance (YouTube comment) is labelled twice, possibly with inconsistent labels. To avoid data leakage between training and testing splits in cross validation, we use 8-fold cross validation where in each fold we use all the comments annotated by one annotator as a test set. We report the performance of the trained models using the same measures as are used for the annotator agreement: Krippendorff’s Alpha-reliability ($Alpha$)^41^, accuracy ($Acc$), and the $F_{1}$  score for individual classes, on both the training and the evaluation datasets. The validation results are reported in Table 3. The coincidence matrices for the evaluation set, used to compute all the scores of the annotator agreements and the model performance, are reported in Table S8 of SI.

**Table 3:** Performance of our hate speech classification model on the training set (cross validation results) and the out-of-sample evaluation set, in comparison to the inter-annotator agreement on the same datasets. The overall performance is measured by Krippendorff’s $Alpha$ and accuracy ($Acc$), and performance for individual classes by $F_{1}$. Note that the performance of our model is comparable to the annotator agreement, except for the Violent class, indicated by lower $F_{1}$.

Performance and agreement	Overall		Acceptable	Inappropriate	Offensive	Violent
	$Alpha$	$Acc$	$F_{1}$	$F_{1}$	$F_{1}$	$F_{1}$
**Model**						
Training	0.59	0.79	0.87	0.54	0.64	0.52
Evaluation	0.55	0.84	0.91	0.59	0.58	0.39
**Inter-annotator**						
Training	0.59	0.77	0.86	0.52	0.63	0.63
Evaluation	0.56	0.82	0.90	0.53	0.57	0.55

The performance of our model is comparable to the annotator agreement in terms of Krippendorff’s $Alpha$  and accuracy ($Acc$), providing evidence for its high quality. The model achieves the annotator agreement both on the training set in the cross validation setting, as well as on the evaluation set. This shows the ability of the model to generalise well on the yet unseen, out-of-sample evaluation data. We observe similar results in terms of $F_{1}$  scores for individual classes. The only noticeable drop in performance compared to the annotators is the performance on the minority (Violent) class. We attribute this drop to the very low amount of data available for the Violent class compared to the other classes, however, the performance is still reasonable. We therefore apply our hate speech detection model to the set of 1.3M comments and report the findings.

## Results and discussion

### Relationship between hate speech and misinformation

**Figure 2:**
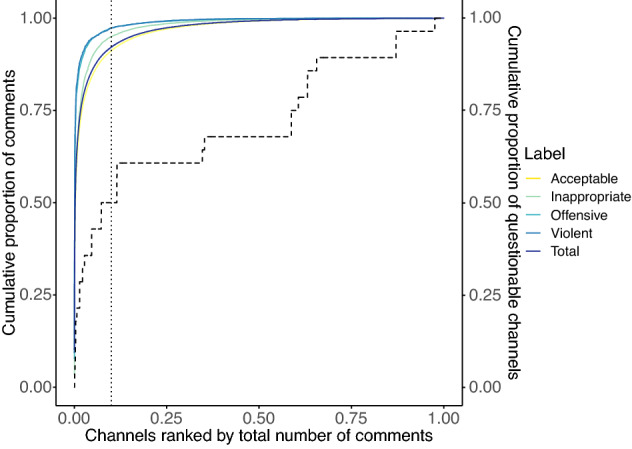
Ranking of YouTube channels by number of comments and proportions of comment types per channel.

We start our analysis examining the distribution of the different speech types on both reliable and questionable YouTube channels. Figure 2 shows the cumulative distribution of comments, total and per type, by channel. The x-axis shows the YouTube channels ranked by their total number of comments, while the y-axis shows the total number of comments in the dataset (both quantities are reported as proportions). We observe that the distribution of comments is Pareto-like; indeed, the first 10% of channels (dotted vertical line) covers about 90% of the total number of comments. Such a 10 to 90 percent relationship is even stronger when comments are analysed according to their types; indeed, the heterogeneity of the distribution decreases going from violent to acceptable comments. It is also worth noting that, as indicated by the secondary y-axis of Fig. 2, the first 10% of channels with most comments also contain about 50% of all the questionable channels in our list, thus indicating a relatively high popularity of these channels. In addition, questionable channels are about 0.25% of the total number of channels that received at least one comment and, despite being such a minority, they cover $\sim$ 8% of the total number of comments (with the following partitioning: 8% acceptable; 7% inappropriate; 9% offensive; 9% violent) and the 1.3% of the total number of videos, thus highlighting a disproportion between their activity and popularity.

**Figure 3:**
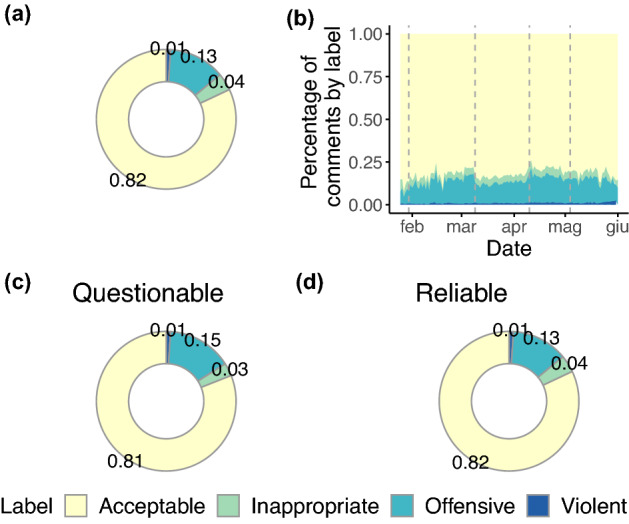
Proportion of the four hate speech labels in the whole dataset (**a**) over time (**b**), and for questionable (**c**) and reliable (**d**) YouTube channels. Panel b displays four dashed lines in correspondence of events of paramount relevance for the year 2020 in Italy. The first line is placed on 30/01/2020 when the first two cases of COVID-19 were detected in Italy. The second line is placed on 09/03/2020 when the Prime Minister enforced the first lockdown to the whole nation. The third line is placed on 10/04/2020 when the Prime Minister communicated to the nation an extension of the lockdown until May the 3rd. The fourth line is placed on 04/05/2020 when the “phase 2” (i.e., the suspension of the full lockdown) began. Interestingly, we note a higher share of Acceptable comments between the second and third lines, that is during the lockdown, perhaps due to positive messages and encouragement among people. Instead, as a possible consequence of the extension of the lockdown, we note a lower share of Acceptable comments right after the third line.

Figure 3 shows the proportion of comments by label and channel types, and their trend over time. In panel (a) we display the overall proportion of comment types, noting that the majority of comments is acceptable, followed by offensive, inappropriate, and violent types, all relatively stable over time (see panel (b)). It is worth remarking that, despite the proportion of hate speech found in the dataset is consistent with—although slightly higher than—previous studies^20,33^, the presence of even a limited number of hateful comments is in direct conflict with the platform’s policy against hate speech. Moreover, we do not observe relevant differences between questionable (panel (c)) and reliable (panel (d)) channels, providing a first piece of evidence in favour of a moderate (if not absent) relationship between online hate and misinformation.

**Figure 4:**
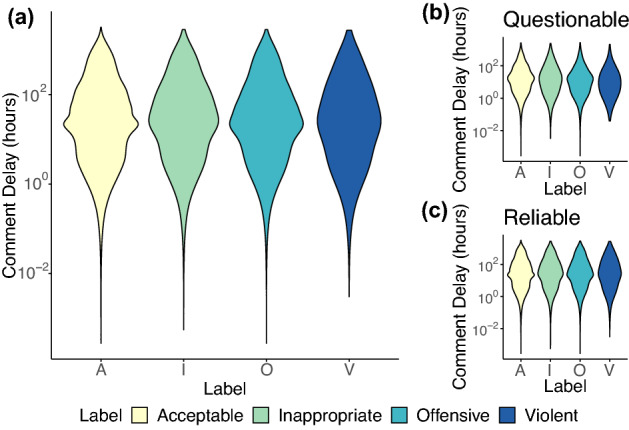
Distribution of comment delays in the whole dataset (**a**) and for questionable (**b**) and reliable (**c**) YouTube channels. The capital letters on the x-axis represent the different types of comments: acceptable (A); inappropriate (I); offensive (O); violent (V).

Now we aim at understanding whether hateful comments display a typical (technically, the average) time of appearance. This kind of information can indeed be crucial for the implementation of timely moderation efforts. More specifically, our goal is to discover whether 1) different speech types have typical delays and 2) any difference holds between comments on videos disseminated by questionable and reliable channels. To this aim, we define the comment delay as the time elapsed between the posting time of the video and that of the comment (in hours). Figure 4 displays the comment delays for the four types of hate speech and for questionable and reliable channels. Looking at panel (a) of Fig. 4, we first note that all comments share approximately the same delay regardless of their type. Indeed, the distributions of the comment delay are roughly log-normal with a long average delay ranging from 120 h in the case of acceptable comments to 128 h in the case of violent comments (the comment delay is reduced by $\sim 75\%$ when removing observations in the right tail of the distribution as shown in Table S1 of SI). For what concerns comments on videos published by questionable and reliable channels, we do not find strong differences between typical delays of speech types within the two domains. In the case of questionable channels, we find that comment delays range from 66 to 42 h, while for reliable channels they range from 125 to 136 h (as reported in SI). To summarise, we find a discrepancy in users’ responsiveness to the two types of content, with comments on questionable videos having a much lower typical delay than those on reliable videos. In addition, comments typical delays differ between reliable and questionable channels. In particular, on questionable channels toxic comments appear first and faster than acceptable ones, following decreasing levels of toxicity (violent $\rightarrow$ offensive $\rightarrow$ inappropriate). In other words, violent comments on questionable content display the shortest typical delay, followed by offensive, inappropriate, and acceptable comments. Conversely, on reliable channels the shortest typical delay is observed for appropriate comments, followed by violent, unacceptable, and offensive comments (for details refer to SI).

### Users’ behaviour and misinformation

**Figure 5:**
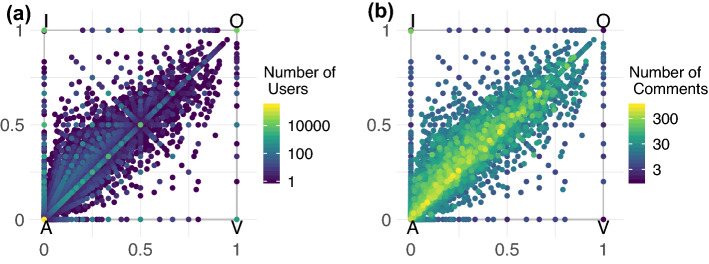
Users balance between different comment types. In panel (**a**) brighter dots indicate a higher density of users while in panel (**b**) brighter dots indicate a higher average activity of the users in terms of number of comments. We note that users focused on posting comments labelled as offensive and violent are almost absent in the data.

In line with other social media platforms^30,46^, users activity on YouTube follows a heavy tailed distribution, i.e., the majority of users post few comments, while a small minority is hyperactive (see Fig. S1 of SI for details). Now we want to investigate whether a systematic tendency towards offences and hate can be observed for some (category of) users. In Fig. 5, each vertex of the square represents one of the four speech types (acceptable—A; inappropriate—I; offensive—O; violent—V). Each dot is a user whose position in the square depends on the fraction of his/her comments for each category. As an example, a user posting only acceptable comments will be located exactly on the vertex A (i.e., in (0,0)), while a user that splits his/her activity evenly between acceptable and inappropriate comments will be located in the middle of the edge connecting the vertices A and I. Similarly, a user posting only violent comments will be located exactly on the vertex V (i.e., in (1,0)). More formally, to shrink the 4-dimensional space deriving by the four labels that fully characterise the activity of each user, we associate a user *j* the following coordinates in a 2-dimensional space:

1 $$\begin{aligned} x_j= a_j*0 + i_j*0 + o_j*1 + v_j*1 \end{aligned}$$

2 $$\begin{aligned} y_j= a_j*0 + i_j*1 + o_j*1 + v_j*0 \end{aligned}$$

where $a_j$, $i_j$, $o_j$, $v_j$ are the proportions, respectively, of acceptable, inappropriate, offensive, and violent comments posted by user *j* over his/her total activity $c_j$.

Although most of the users leave only or mostly acceptable comments, there are also several users ranging across categories (i.e., located away from the vertices of the square in Fig. 5). Interestingly, there is no evidence of “pure haters”, i.e., active users exclusively using hateful language, that are only 0.3% of the total number of users. Indeed, while there are users posting only or mostly violent comments (see Fig. 5a), their overall activity is very low and below five comments (see Fig. 5b). A similar situation is observed for offenders, i.e., active users posting only offending comments. Although we cannot exclude that moderation efforts put in place by YouTube (if any) might partially impact these results, the absence of pure haters and offenders highlights that hate speech is rarely only an issue of specific categories of users. Rather, it seems that regular users are occasionally triggered by external factors. To rule out possible confounding factors (note that users located in the centre of the square could display a balanced activity between different pairs of comment categories) we repeated the analysis excluding the category I (i.e., inappropriate). The results are provided in SI and confirm what we observe in Fig. 5.

We now aim at unveiling the relationship between users behaviour in terms of commenting patterns and their activity with respect to questionable and reliable channels. Since misinformation is often associated with the diffusion of polarising content which plays on one’s fear and could fuel anger, frustration and hate^47–49^, our intent is to understand whether users more loyal to questionable content are also more prone to use a toxic language in their comments. Thus, we define the leaning *l* of a user *j* as the fraction of his/her activity spent in commenting videos posted by questionable channels, i.e.,

3 $$\begin{aligned} l_j = \sum _{i = 1}^{c_j}\frac{q_j}{c_j} \end{aligned}$$

where $\sum _{i = 1}^{c_j}q_j$ is the number of comments on videos from questionable channels posted by the user *j* and $c_j$ is the activity of user *j*. Similarly, for each user *j* we compute the fraction of unacceptable comments ${\overline{a}}$ as:

4 $$\begin{aligned} {\overline{a}}_j = 1 - a_j \end{aligned}$$

where $a_j$ is the fraction of acceptable comments posted by user *j*.

**Figure 6:**
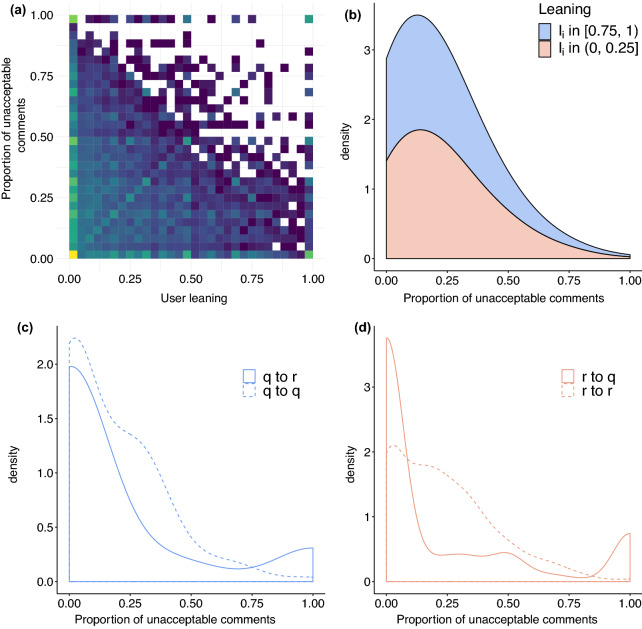
Panel (**a**) displays the relationship occurring between the preference of users for questionable and reliable channels (the user leaning $l_j$) and the fraction of unacceptable comments posted by the user (${\overline{a}}_j$) as a joint distribution. Panel (**b**) displays the distribution of unacceptable comments for users displaying a remarkable tendency to comment under videos posted by questionable ($l_j \in [0.75,1)$) and reliable ($l_j \in (0,0.25]$) channels. Panel (**c**) displays the distribution of unacceptable comments posted by users with leaning towards questionable channels ($l_j \in [0.75,1)$ indicated as q) under videos of questionable channels (dashed line indicated as q to q in the legend) and under videos of reliable channels (solid line indicated as q to r in the legend). Panel (**d**) displays the distribution of unacceptable comments posted by users with leaning towards reliable channels ($l_j \in (0,0.25]$ indicated as r) under videos of questionable channels (solid line indicated as r to q in the legend) and under videos of reliable channels (dashed line indicated as r to r in the legend).

In Fig. 6a, we compare users’ leaning $l_j$ against the fraction of unacceptable comments ${\overline{a}}_j$. As expected, we may observe two peaks (of different magnitude) in correspondence of extreme values of leaning ($l_j \sim 0$ and $l_j \sim 1$), represented by the brighter squares in the plot. In addition, the joint distribution becomes sparser in correspondence of higher values of users’ leaning and fraction of unacceptable comments ($l_j \ge 0.5$ and ${\overline{a}}_j \ge 0.5$), indicating that a relevant share of users are placed at the two extremes of the distribution (thus being somewhat polarised) and that users producing mostly unacceptable comments are way less present.

In Fig. 6b, we display the proportion of unacceptable comments posted by users displaying leaning at the two tails of the distribution (i.e., users displaying a remarkable tendency to comment questionable videos $l_j \in [0.75,1)$ and users with a remarkable tendency to comment reliable videos $l_j \in (0,0.25]$). We find that users skewed towards reliable channels post, on average, a higher proportion of unacceptable comments ($\sim 23\%$) than users skewed towards questionable channels ($\sim 17\%$). In other words, users who tend to comment on reliable videos are also more prone to use a unacceptable/toxic language. Further statistics on the two distributions are reported in SI.

Panel (c) of Fig. 6 provides a comparison between the distributions of unacceptable comments posted by users skewed towards questionable channels (*q* in the legend) on videos published by either questionable or reliable channels. Panel (d) of Fig. 6 provides a similar representation for users skewed towards reliable channels (*r* in the legend). We may note a strong difference in users behaviour: quite unimodal when they comment videos on the same side of the leaning; bimodal when they comment videos on the opposite side of leaning. Therefore, users tend to avoid using a toxic language when they comment videos in accordance with their leaning and to separate into roughly two classes (non-toxic, toxic) when they comment videos in contrast with their preferences. This finding resonates with evidence of online polarisation and with the presence of peculiar characters of the internet such as trolls and social justice warriors.

### Toxicity level of online debates

Finally, we aim at investigating whether online debates degenerate (i.e., increase their average toxicity) when the discussion gets longer, both in terms of number of comments and time. More in general, we are interested in analysing how commenting dynamics change over time and whether online hate follows similar dynamics to those observed for users’ sentiment^31^. Indeed, although violent comments and pure haters are quite rare, their presence could negatively impact the tone of the general debate. Furthermore, we want to understand whether the toxicity of comments tends to follow certain dynamics empirically observed on the internet such as Godwin’s law. To this purpose, we test whether toxic comments tend to appear more frequently at later stages of the debate.

To compute the toxicity level of a debate around a certain video, we assign each speech type (A,I,O,V) a toxicity value *t* as follows:

- Acceptable: *t* = 0
- Inappropriate: *t* = 1
- Offensive: *t* = 2
- Violent: *t* = 3

Then, we define the toxicity level *T* of a discussion *d* of *n* comments as the average of the toxicity values over all the comments of the discussion:

$$\begin{aligned} T_d = \frac{\sum _{j=1}^{n}{t_j}}{n}. \end{aligned}$$

**Figure 7:**
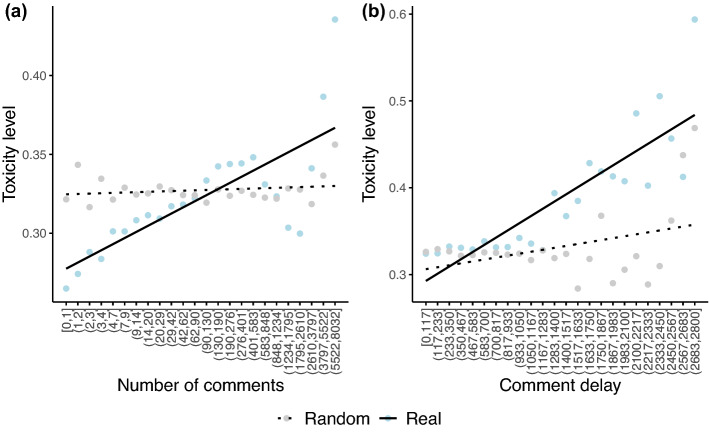
Linear regression models for number of comments and comment delay. On the x-axis of panel (**a**) the comments are grouped in logarithmic bins while on the x-axis of panel (**b**) the comment delays are grouped in linear bins.

To understand how the toxicity level changes with respect to the number of comments and to comment delay (i.e., the time elapsed between the posting time of the video and that of the comment), we employ linear regression models. Figure 7 shows that a positive relationship between the two variables (i.e., average toxicity is an increasing function of the number of comments and comment delay) exists, and that such a relationship cannot be reproduced by linear models obtained with randomised comment labels (regression outcomes and a validation of our results using proportions of unacceptable comments are reported in SI). We apply a similar approach to distinguish between comments on videos from questionable and reliable channels (as shown in SI). Overall, similarly to the general case, we find stronger positive effects in real data than in randomised models although such effects are significant only in the case of comments under videos posted by reliable channels.

**Figure 8:**
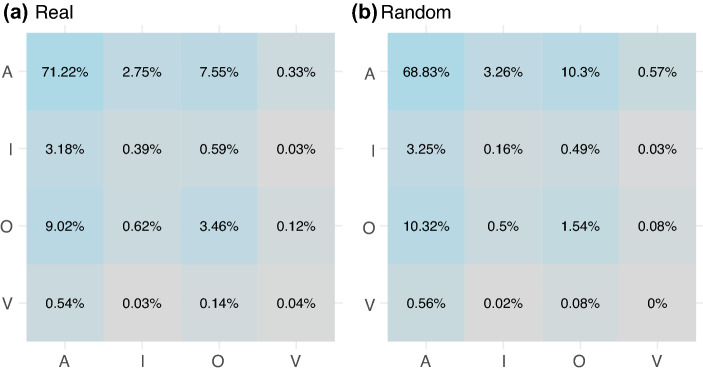
Transition probabilities between different comments types represented by a $4 \times 4$ transition matrix in the real (panel **a**) and in the random case (panel **b**). Brighter entries of the matrix indicate higher transition probabilities.

Finally, to evaluate the effect (in the short run) of violent comments, we study the transition between subsequent comments in threads appearing under YouTube videos. The choice of analysing threads instead of full lists of comments resides in the fact that YouTube comments are ranked according to several factors (among which the number of likes received by the comment, the length of the thread, the importance of the user who posted the comment). Therefore, given a certain video, we cannot be sure of what comments (and in which order) the user actually visualises. However, threads do not suffer from this issue, since comments in threads are presented in chronological order. The aim of studying the transitions between comment types is to find specific transition patterns (probabilities) between toxic comments and understand if the conversation tends to evolve in a way that is different with respect to random models. As an example, a thread with four comments 1 Acceptable, 2 Offensive and 1 Violent (in this order) can be summarised with the string “AOOV”, which entails three transitions between comment types, namely {AO; OO; OV}. By extending such a process to all threads in our dataset, we can compute the transition probability from one comment type to another using a 4 by 4 transition matrix. In this way, we can evaluate the possible presence of an escalation effect due to the fact that toxic comments could be immediately followed by increasingly toxic ones. The results are reported in Fig. 8, in which we notice that certain transition probabilities cannot be reproduced by a null model in which the sequences of comments within threads are randomised. In particular, we note that, differently from the empirical data, in randomised instances the transition probability from one violent comment to another is 0 and the probability of passing from violent comments to unacceptable ones (inappropriate, offensive and violent) is always higher in the empirical case than at random. Similar results hold for offensive and inappropriate comments, but not for acceptable ones. This finding confirms the presence of a short term influence of violent comments that could flame the debate and scale up into streams of toxicity.

## Conclusions

The aim of this work is two-fold: i) to investigate the behavioural dynamics of online hate speech and ii) to shed light on the possible relationship with misinformation exposure and consumption. We apply a hate speech deep learning model to a large corpus of more than one millions comments on Italian YouTube videos. Our analysis provides a series of important results which can support the development of appropriate solutions to prevent and counter the spread of hate speech online. *First*, there is no evidence of a strict relationship between the usage of a toxic language (including hate speech) and being involved within the misinformation community on YouTube. *Second*, we do not observe the presence of “pure” haters, instead it seems that the phenomenon of hate speech involves regular users who are occasionally triggered to use toxic language. *Third*, users polarisation and hate speech seem to be intertwined, indeed users are more prone to use inappropriate, violent, or hateful language within their opponents community (i.e., out of their echo chamber). *Finally*, we find a positive correlation between the overall toxicity of the discussion and its length, measured both in terms of number of comments and time.

Our results are in line with recent studies about (the increasing) polarisation of online debates and segregation of users^50^. Furthermore, they somewhat confirm the intuition behind some empirically grounded laws such as Godwin’s law which can be interpreted, by extension, as a statement regarding the increasing toxicity of online debates. A potential limitation of this work is represented by the relentless effort of YouTube in moderating hate on the platform. This could have prevented us from having complete information about the actual presence of hate speech in public discussions. In spite of this limitation, after collecting again the whole set of comments after at least 1 year from their posting time, we find that only 32% of violent comments were actually unavailable due to either moderation or removal by the author (see Table S9 of SI). Another issue could be the presence of channels wrongly labelled as reliable instead of questionable (i.e., false negatives) or the fact that certain questionable sources available on YouTube are not included in the list, especially due to the high variety of content available on the platform and the relative ease with which one can open a new channel. Nonetheless, our findings are robust with respect to these aspects (as we show in a dedicated section of SI). Future efforts should extend our work to other languages beyond Italian, social media platforms, and topics. For instance, studying hate speech on online political discourse over time could provide important insights on debated phenomena such as affective polarisation^51^. Moreover, further research on possible triggers in the language and content of videos is desirable.

## Supplementary Information

Supplementary Table S1.

## Data Availability

The datasets generated during the current study for the purposes of training and evaluating the hate speech model are available at the CLARIN repository: http://hdl.handle.net/11356/1450. The hate speech model is available at the HuggingFace repository: https://huggingface.co/IMSyPP/hate_speech_it.
