# Supplementary material for: Dynamics of online hate and misinformation
Source: Sci Rep. 2021 Nov 11;11:22083. doi: 10.1038/s41598-021-01487-w (PMC8585974; doi:10.1038/s41598-021-01487-w)
Supplement: Supplementary file 1 — Supplementary Table S1. [file 41598_2021_1487_MOESM1_ESM.pdf]

Supplementary Information

	μ_{cd}	σ_{cd}	γ_{cd}	$20\%\mu_{cd}$
Acceptable	120	259	3.85	35.4
Inappropriate	128	269	3.77	39.7
Offensive	128	278	3.84	36.8
Violent	127	272	3.87	38.2

Table S1: Comment delays in hours for each category. The first column displays the average comment delay (μ_{cd}); the second column displays the standard deviation of the comment delay (σ_{cd}); the third column displays the skewness (γ_{cd}), i.e., the asymmetry of the comment delays that is always positive thus indicating the presence of deviations in the right tail; the fourth column displays the 20% trimmed mean ($20\%\mu_{cd}$), i.e., the average comment delay after removing the 20% of comments having the highest comment delays.

	$\hat{\mu}_{cd}$		$\hat{\sigma}_{cd}$	
	Questionable	Reliable	Questionable	Reliable
Acceptable	65.9	124.6	7.4	6.7
Inappropriate	65.5	132.2	7.3	7.1
Offensive	53.3	135.0	9.1	6.9
Violent	41.7	135.9	7.8	7.4

Table S2: Comment delays in hours for each category of comments and channels. Since the two categories have different sample size the summary statistics reported in this table derive from a bootstrap of 7,500 samples per category repeated 1,000 times. The first column displays the average bootstrap comment delay ($\hat{\mu}_{cd}$); the second column displays the standard deviation of the average bootstrap comment delay ($\hat{\sigma}_{cd}$).

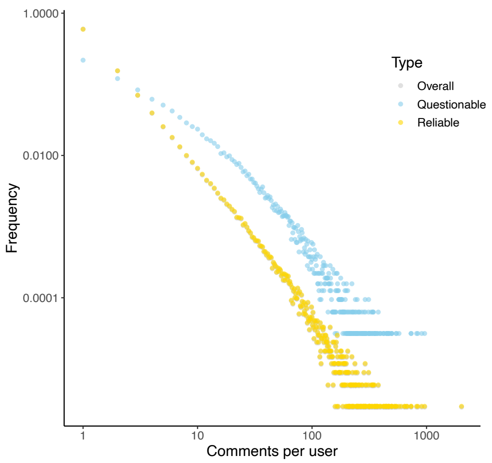

Figure S1: Distribution of comments per user in the general case and restricting the count only to comments posted by the user under either questionable or reliable videos.

	$\mu_{\overline{a}}$	$\sigma_{\overline{a}}$	$\gamma_{\overline{a}}$
$l_j \in (0, 0.25]$	0.23	0.19	0.94
$l_j \in [0.75, 1)$	0.17	0.20	1.29

Table S3: Summary statistics of proportions of unacceptable comments by users with leaning skewed towards reliable ($l_j \in (0, 0.25]$) and questionable ($l_j \in [0.75, 1)$) channels. The first column displays the average proportion of unacceptable comments ($\mu_{\overline{a}}$); the second column displays the standard deviation of the proportion of unacceptable comments ($\sigma_{\overline{a}}$); the third column displays the skewness ($\gamma_{\overline{a}}$), i.e., the asymmetry of the distributions, that is positive thus indicating the presence of deviations in the right tail.

Robustness Check

Although the AGCOM source list aims to be as comprehensive as possible, it is reasonable to assume that some of the questionable sources available on YouTube are not included in the list, especially due to the high variety of content available on the platform and the relative ease with which one can open a new channel. Our empirical results show that the distribution of hate speech types is similar in questionable and reliable channels (see Figure 3) and that users skewed towards reliable channels use, on average, a more toxic language than those skewed towards questionable channels (see panel b of Figure 6). To validate these findings, we simulated several scenarios. In each simulated scenario, we assign a certain percentage of reliable channels (possibly containing false negatives) to the list of questionable channels (true positives) listed in the AGCOM source. We assume the false negative rates (i.e., percentages of

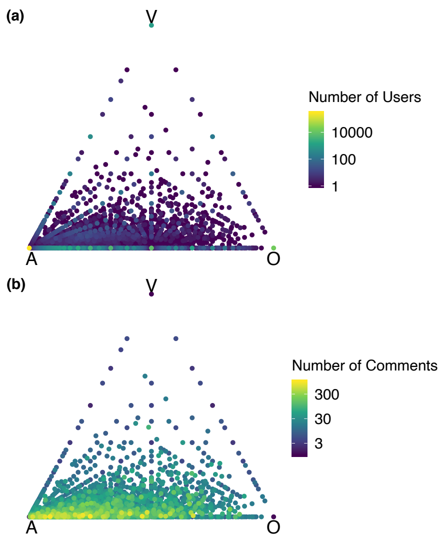

Figure S2: Users balance between different comment types. Comments labelled as inappropriate (I) are eliminated with respect to Figure 5. Each dot is mapped into the triangle using barycentric coordinates. In panel (a) brighter dots indicate a higher density of users while in panel (b) brighter dots indicate a higher average activity of the users in terms of number of comments. Consistently with Figure 5, we note that users focused on posting comments labelled as offensive and violent are almost absent in the data.

channels wrongly labelled as reliable) to vary in the set {0.1%, 0.5%, 1%, 5%, 15%, 30%}, corresponding, respectively, to {7, 36, 71, 357, 1071, 2142} additional questionable channels, and we reproduce our results 1,000 times. It is reasonable to limit the size of questionable channels to 30%, since the fraction of misinformation-related channels on social media platforms may range up 30% of the total, as shown for instance in [11]. The Figure S5 below shows the distributions of the difference (called Delta) between the percentages of the four comment types found under videos posted by questionable and reliable channels. The distributions are displayed for different false negative rates and each distribution is made up of 1,000 samples. Specifically, we note that, despite correcting for different false negative rates, all the distributions of Delta are peaked around zero. This is in line with what shown in Figure 3 (in the main paper), i.e., we do not find prominent differences in the percentages of hate speech types between questionable and reliable channels.

By using an akin approach, we present the distributions of the percentage of unacceptable comments posted by users skewed towards questionable and reliable channels. The separation between the distributions in Figure S6 suggests that users skewed towards reliable channels ($l_i \in (0, 0.25]$) post, on average, a higher number of unacceptable comments than users skewed towards question-

	Model 1 Number of comments (Real)	Model 2 Number of comments (Random)	Model 3 Comment delay (Real)	Model 4 Comment delay (Random)
(Intercept)	0.2736^{***}	0.3244^{***}	0.2844^{***}	0.3040^{***}
	(0.0101)	(0.0036)	(0.0158)	(0.0167)
x	0.0039^{***}	0.0002	0.0083^{***}	0.0022
	(0.0007)	(0.0003)	(0.0011)	(0.0012)
R^2	0.5768	0.0365	0.7188	0.1414
Adj. R^2	0.5576	−0.0073	0.7061	0.1024

$^{***}p < 0.001$; $^{**}p < 0.01$; $^{*}p < 0.05$

Table S4: Statistical models of Figure 7 a and b.

	Model 1 Questionable (Real)	Model 2 Questionable (Random)	Model 3 Reliable (Real)	Model 4 Reliable (Random)
(Intercept)	0.3256^{***}	0.3285^{***}	0.2704^{***}	0.3243^{***}
	(0.0214)	(0.0084)	(0.0110)	(0.0036)
x	0.0007	−0.0004	0.0041^{***}	0.0003
	(0.0016)	(0.0006)	(0.0008)	(0.0002)
R^2	0.0093	0.0190	0.5589	0.0474
Adj. R^2	−0.0379	−0.0277	0.5388	0.0041
Num. obs.	23	23	24	24

$^{***}p < 0.001$; $^{**}p < 0.01$; $^{*}p < 0.05$

Table S5: Statistical models for number of comments. See panels (a) and (b) of Figure S4.

able channels ($l_i \in [0.75, 1)$).

To further stress the latter finding we also perform a bootstrap analysis. Indeed, the average proportions of unacceptable comments observed in empirical data could be biased by the unbalance between the two types of users, 837 skewed towards questionable channels ($l_i \in [0.75, 1)$) and 11,215 skewed towards reliable ones ($l_i \in (0, 0.25]$). For this reason, we sampled the average proportion of unacceptable comments posted by 837 users skewed towards reliable channels 1,000 and we compared the distribution of such average values with the average proportion of unacceptable comments posted by users skewed towards questionable channels in empirical data. Even in this case, as shown in Figure S7, we find a clear-cut difference between the two types of users.

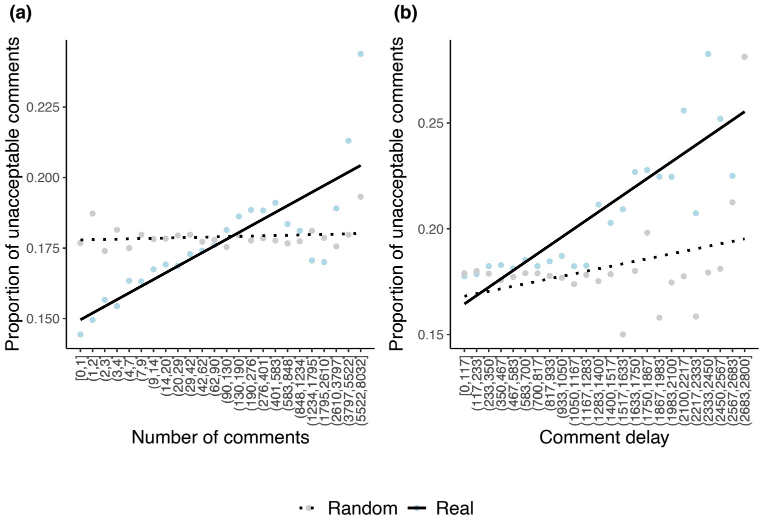

Figure S3: Linear regression models of proportions of unacceptable comments for number of comments and comment delay. On the x-axis of panel (a) the comments are grouped in logarithmic bins while on the x-axis of panel (b) the comment delays are grouped in linear bins.

	Model 1 Questionable (Real)	Model 2 Questionable (Random)	Model 3 Reliable (Real)	Model 4 Reliable (Random)
(Intercept)	0.1619	0.3337***	0.2900***	0.3059***
	(0.1552)	(0.0822)	(0.0142)	(0.0198)
x	0.0165	0.0023	0.0078***	0.0018
	(0.0109)	(0.0058)	(0.0010)	(0.0014)
R^2	0.0950	0.0070	0.7359	0.0746
Adj. R^2	0.0539	−0.0381	0.7239	0.0326
Num. obs.	24	24	24	24

$^{***}p < 0.001$; $^{**}p < 0.01$; $^{*}p < 0.05$

Table S6: Statistical models for comments delay. See panels (c) and (d) of Figure S4.

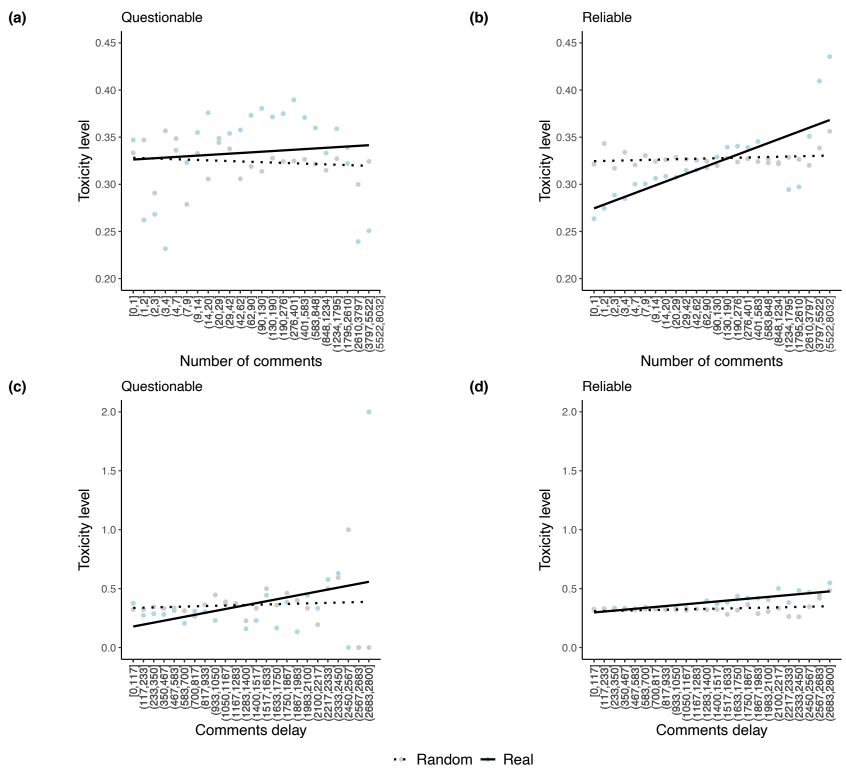

Figure S4: Linear regression models for number of comments and comment delay separating videos posted by questionable (panels (a) and (c)) and reliable channels (panels (b) and (d)). On the x-axis of panel (a) and (b) the comments are grouped in logarithmic bins while on the x-axis of panel (c) and (d) the comment delays are grouped in linear bins.

Example	Translation	Annot. 1	Annot. 2	Model
L'Italia è una grande nazione così come il suo popolo, sono i politici che abbiamo che sono parassiti corrotti, il paese è da ripulire da tutti questi parassiti con un disinfestante.	Italy is a great nation, just like its people, it's our politicians that are corrupt parasites, we need to clear the country of these parasites with a disinfectant.	Violent	Offensive	Violent
Ma datevi fuoco, siete 4 FESSI.	Set yourselves on fire, you are 4 dumbasses.	Violent	Violent	Violent
Rimetteteli subito in carcere e gettate le chiavi in mare	Lock them up and throw the keys into the sea	Acceptable	Violent	Acceptable
Per questo doveva stare anche lui doveva marcire lì dentro, non è che poi uno si pente e diventa un santo	For this he needed to stay as well, he needed to march in, you cannot just repent after the fact and become a saint	Violent	Offensive	Acceptable
Le solite porcate Italiane.. Per cambiare l'italia ci vogliono 7 Hiroshima	The usual Italian shenanigans ... 7 Hiroshima's are needed to change Italy	Violent	Inappropriate	Acceptable

Table S7: A sample of YouTube comments, with labels assigned by the annotators and the model. In the top two cases (examples 1 and 2) the annotators and the model (mostly) agree. There are three common cases where the model systematically disagrees with at least one of the annotators. In the first case (example 3) the annotators also systematically disagree with one another, confirming our assumption that hate speech is a difficult task for human annotators. In the second case (example 4), the labelled tweet is not hateful on its own, however both annotators mark it as violent or offensive. Since the annotators had access to the whole threads during annotation, this suggests that the hatefulness of the message stems from the missing context. In the third case (example 5) we have the presence of metaphorical language; the model fails to recognise that the word "Hiroshima" refers to nuclear weapons. This is a common, but difficult to solve problem for computational models, and is subject of ongoing research.

Label	A	I	O	V	$\sum$
A	14,330	127	1,444	49	15,950
I	127	408	227	8	770
O	1,444	227	2,342	62	4,075
V	49	8	62	144	263
$\sum$	15,950	770	4,075	263	21,058

Label	A	I	O	V	$\sum$
A	22,900	189	1,824	114	25,027
I	189	670	265	11	1,135
O	1,824	265	2,946	75	5,110
V	114	11	75	130	330
$\sum$	25,027	1,135	5,110	330	31,602

Table S8: Coincidence matrices for the evaluation set: coincidences between two different annotators (top), and coincidences between the annotators and the model, but not between the annotators themselves (bottom). The grand totals for each matrix represent the number of pairable labels over all the comments. Note that a small fraction of comments was not annotated twice, therefore there are 21,058 pairs in the top matrix, instead of 21,086. All performance measures, Krippendorff's $Alpha$, Acc, and F_1 are calculated from these matrices. The axes show the possible labels (A = Acceptable, I = Inappropriate, O = Offensive, V = Violent).

Label	Removed	n	Percentage
Acceptable	FALSE	879,871	84.0
Acceptable	TRUE	167,193	16.0
Inappropriate	FALSE	41,400	81.3
Inappropriate	TRUE	9,549	18.7
Offensive	FALSE	130,963	79.6
Offensive	TRUE	33,637	20.4
Violent	FALSE	7,703	68.0
Violent	TRUE	3,628	32.0

Table S9: Percentage of comments in the dataset after collecting again the whole comment set of comments with at least one year of delay with respect to their posting time. We note that increasingly toxic comments have a higher probability to be removed.

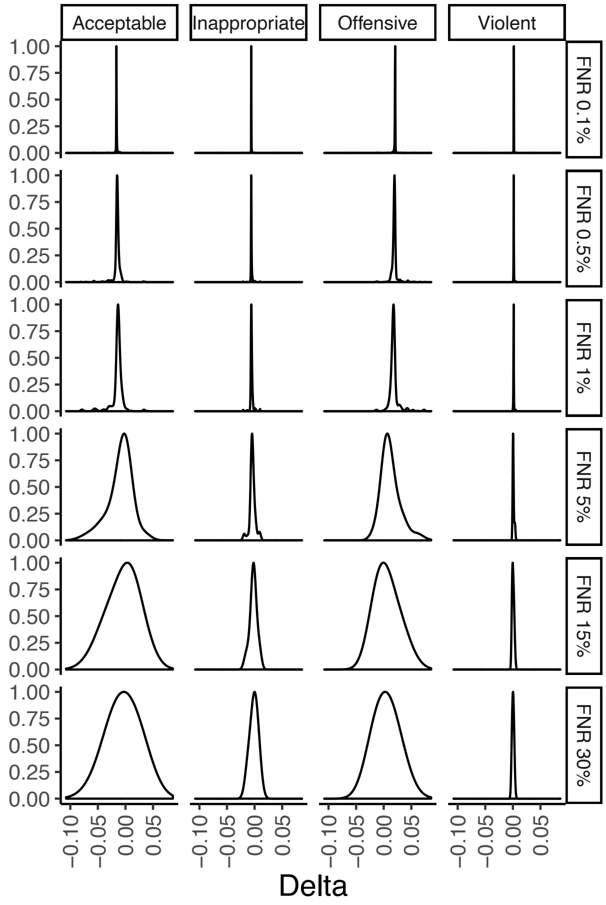

Figure S5: Distribution of the difference between comment types posted under videos by questionable and reliable channels for different false negatives rates.

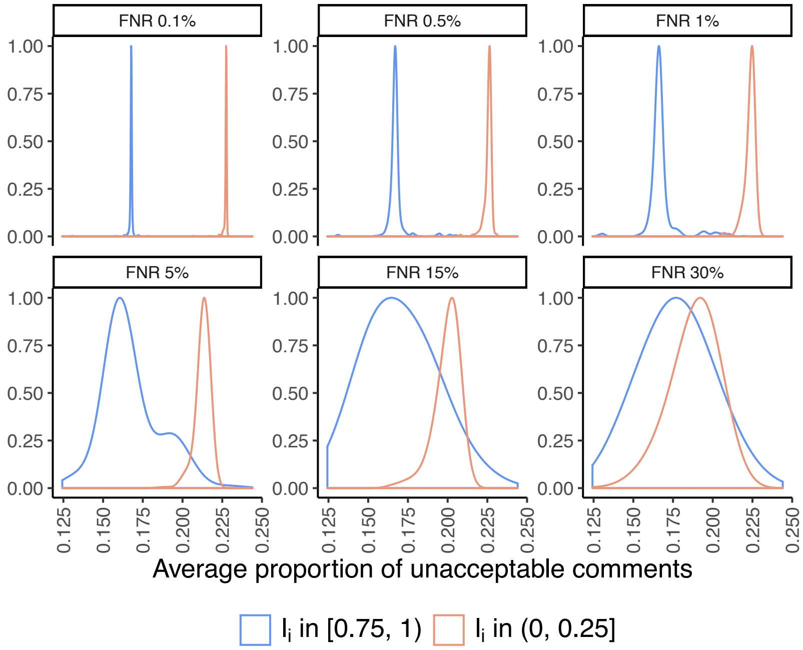

Figure S6: Distributions of the average proportions of unacceptable comments for users skewed towards questionable and reliable channels.

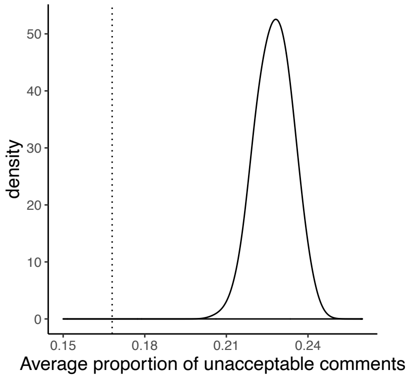

Figure S7: The distribution refers to the average proportion of unacceptable comments posted by users skewed towards reliable channels ($l_i \in (0, 0.25]$) across 1,000 samples while the dashed line indicates the same proportion but posted by users skewed towards questionable channels ($l_i \in [0.75, 1)$).
